# Adverse Events and Immunogenicity of mRNA-Based COVID-19 Vaccine among Healthcare Workers: A Single-Centre Experience

**DOI:** 10.3390/medicina58030441

**Published:** 2022-03-17

**Authors:** Jolanta Sauserienė, Ida Liseckienė, Vitalija Neverauskė, Eglė Šepetauskienė, Danielius Serapinas, Šarūnas Mačinskas, Brigita Šitkauskienė, Ieva Bajoriūnienė, Rūta Vaičiūnienė, Leonas Valius

**Affiliations:** 1 Department of Family Medicine, Medical Academy, Lithuanian University of Health Sciences, LT-44307 Kaunas, Lithuania; ida.liseckiene@kaunoklinikos.lt (I.L.); vitalija.neverauske@kaunoklinikos.lt (V.N.); danielius.serapinas@lsmu.lt (D.S.); sarunas.macinskas@kaunoklinikos.lt (Š.M.); leonas.valius@lsmuni.lt (L.V.); 2 Information Technology Centre, Lithuanian University of Health Sciences, LT-44307 Kaunas, Lithuania; egle.sepetauskiene@lsmuni.lt; 3 Department of Immunology and Allergology, Medical Academy, Lithuanian University of Health Sciences, LT-44307 Kaunas, Lithuania; brigita.sitkauskiene@kaunoklinikos.lt (B.Š.); ieva.bajoriuniene@kaunoklinikos.lt (I.B.); 4 Department of Nephrology, Medical Academy, Lithuanian University of Health Sciences, LT-44307 Kaunas, Lithuania; ruta.vaiciuniene@kaunoklinikos.lt

**Keywords:** SARS-CoV-2, Pfizer-BioNTech vaccine, rapid antibody test, adverse event, seroconversion

## Abstract

*Background and Objectives*: The safety and effectiveness of vaccines are among the key priorities in COVID-19 pandemic management. Moreover, evidence-based data regarding vaccine safety and immunogenicity can play an important role in building the trust of the community regarding vaccination. The aim of this study was to investigate the safety and immunogenicity of Pfizer-BioNTech vaccine among healthcare workers in one hospital, 21 days after first dose. *Materials and Methods*: This study was conducted in the Hospital of the Lithuanian University of Health Sciences between February and March 2021. Hospital employees who arrived to receive the second dose of the Pfizer-BioNTech vaccine 21 days after the first one were invited to participate in the study: they were asked to complete an anonymous adverse events questionnaire and were offered a SARS-CoV-2 IgG/IgM rapid test. The study was performed at a single point, 21 days after the first dose of the vaccine. *Results*: Data of 4181 vaccine recipients were analysed. The first vaccine dose was associated with a 53.6% incidence of adverse events, mainly local reactions. Adverse events occurred more frequently in younger participants and women. Moderate adverse events were experienced by 1.4% of the vaccine recipients; 6.2% were incapacitated. Of the 3439 participants who performed a rapid IgG test, 94.5% were positive for IgG antibodies after the first vaccine dose. Seroconversion rates were lower in participants older than 47 years. *Conclusions*: Despite 1.4% moderate adverse events, no safety concerns or anaphylaxis were identified. The Pfizer-BioNTech vaccine induced an immune response in the overwhelming majority of recipients after a single dose. Younger participants experienced adverse events and were positive for IgG antibodies more frequently than older counterparts. It is important to mention that this study specifically considered short-term safety and reactions following vaccination and that long-term adverse effects were not investigated in the study. Thus, future research into both long-term adverse reactions and immune system programming is essential.

## 1. Introduction

The COVID-19 pandemic has caused significant morbidity and mortality across the world, as well as social and economic disruption. There is still an urgent global need for continued vaccination with effective and safe vaccines, and collection of data on safety and immune response is essential. According to the World Health Organization (WHO) operational planning guidelines, developed in 2021, it is important to (1) plan active surveillance of specific COVID-19 vaccine-related adverse events; (2) to identify and secure channels of data-sharing mechanisms to share COVID-19 vaccine safety data and findings with relevant regional and international partners [1]. While findings about promoting vaccination in general are useful in the context of the current pandemic, the acceptance and uptake of COVID-19 vaccines present an unprecedented challenge [2].

In Lithuania, vaccination of healthcare workers as a priority group commenced on 27 December 2020. Timely and reliable scientific data on the safety and predicted efficacy of COVID-19 vaccines are essential for effective public vaccination. There is still a huge demand for data regarding reactogenicity of vaccines and a need to measure the intensity of any adverse events experienced, including short-term local and systemic reactions, as well as to analyse vaccine efficacy and immune response among subgroups by age, gender and other characteristics. Evidence-based data can play a role in building the trust of the community regarding vaccination. Behavioural research has shown that vaccine acceptance and uptake can be increased by adopting special strategies such as harnessing social influences (especially from people who are particularly trusted and identified with by members of relevant communities) and increasing motivation (through open and transparent dialogue and communication about uncertainty and risks, including around the safety and benefits of vaccination) [3].

Evaluation of the incapacity of healthcare workers after COVID-19 vaccination has a core importance aiming to reduce shortages in the healthcare workforce. In addition, the importance of maintaining the ability of healthcare personnel to work is in line with the recommendations of the WHO and the International Labour Organization: ‘Health workers should continue to enjoy their right to decent, healthy and safe working conditions in the context of COVID-19. Healthcare personnel refers to persons serving in healthcare settings who have the potential for direct or indirect exposure to patients or infectious materials, including body substances (e.g., blood, tissue, and specific body fluids); contaminated medical supplies, devices, and equipment; contaminated environmental surfaces; or contaminated air [4]. This includes, emergency medical service personnel, nurses, nursing assistants, home healthcare personnel, physicians, technicians, therapists, phlebotomists, pharmacists, dental healthcare personnel, students and trainees, contractual staff not employed by the healthcare facility, and persons not directly involved in patient care, but who could be exposed to infectious agents that can be transmitted in the healthcare setting (e.g., clerical, dietary, environmental services, laundry, security, engineering and facilities management, administrative, billing, and volunteer personnel) [5]. Mitigating these hazards and protecting the health, safety and well-being of health workers requires well-coordinated and comprehensive measures for infection prevention and control, occupational health and safety, health workforce management and mental health and psychosocial support’ [6]. Comparing the data from national adverse event reporting systems with the data from everyday practices, the numbers and severity of adverse events due to COVID-19 vaccination differ. Neither patients nor medical personnel were involved in adverse event reporting mainly due to a lack of adverse event reporting practice: according to the data of the State Medicines Control Agency of Lithuania during the first four months since the beginning of vaccination (from 27 December 2020 to 30 April 2021), only 2579 reports of adverse events were submitted, accounting for 0.27% of the total number of vaccinations (*n* = 955,921) [7]. The existing gap between the data on side effects reported in national official websites and the data from scientific publications emphasizes the need for continuous evaluation of safety and efficacy regarding COVID-19 vaccine, aiming to share evidence-based data with society [8].

Scientific evidence regarding immunity and the monitoring of short-term and long-term immunity to COVID-19 vaccination are crucial in clinical practice. Understanding the magnitude and characteristics of virus-specific cellular responses and titres of antibody responses required to provide protection will guide both vaccine design and public health policies to limit spread [9].

The aim of this study was to investigate the safety and the effectiveness of the Pfizer-BioNTech vaccine among healthcare workers, to evaluate their ability to work after vaccination, and to present and share up-to-date evidence as recommended in the WHO operational planning guidelines.

## 2. Materials and Methods

### 2.1. Study Setting and Participants

The study was conducted in one of the largest Lithuanian medical institutions, the Hospital of the Lithuanian University of Health Sciences Kaunas Clinics, between February and March 2021. At the beginning of healthcare workers’ vaccination in Lithuania against COVID-19, a vaccination centre was established in this hospital, where the vaccination of employees took place. The mRNA-based vaccine (Pfizer-BioNTech, Mainz, Germany) was the first vaccine imported into Lithuania, and all employees of the hospital, as a group at high risk of SARS-CoV-2 infection, were invited for vaccination with this manufacturer’s vaccine, except those who had suffered from COVID-19 or had medical contraindications. All hospital workers were routinely tested by approved PCR tests on a weekly basis, including before vaccination. Thus, the probability that study participants had specific antibodies to the SARS-CoV-2 virus prior to vaccination is minimal.

The study was performed at the vaccination centre on days when hospital employees arrived to receive the second intramuscular injection of the Pfizer-BioNTech vaccine. The vaccination centre offered a rapid qualitative test to detect specific antibodies (immunoglobulin (Ig) G/M) against SARS-CoV-2 (Singclean, Hangzhou, Zhejiang, China) to all vaccine recipients on a voluntary basis; specificity of the test: IgM 97.3%, IgG 96.4%; sensitivity: IgM 95.7%, IgG 91.8%. Participants were informed about the results of this test within minutes. The members of the research team orally invited all employees to participate in the study, and those who agreed completed an anonymous questionnaire.

### 2.2. Study Instrument

An anonymous questionnaire, developed by the researchers’ team, was used. Participants were asked to indicate whether they experienced adverse events within 15 min after the first dose of the vaccine by checking an appropriate box (yes or no). They were also asked if adverse events occurred after leaving the vaccination centre on the first, second, third and fourth, and following days and whether due to those effects they had to refer to a healthcare institution for medical care. Participants reporting adverse events had to indicate their intensity, whether they took medication to alleviate the experienced symptoms and whether they were incapacitated after receiving the first vaccine dose. The intensity of systemic and local adverse events listed in the questionnaire was rated on a scale from 1 (very mild symptoms) to 5 (very severe symptoms). Participants were asked to rate the intensity of adverse events on the first, second, third and fourth, and following days after vaccination separately. It was also permitted to report other symptoms not included in the list by writing them down in the questionnaire.

**Figure 1:**
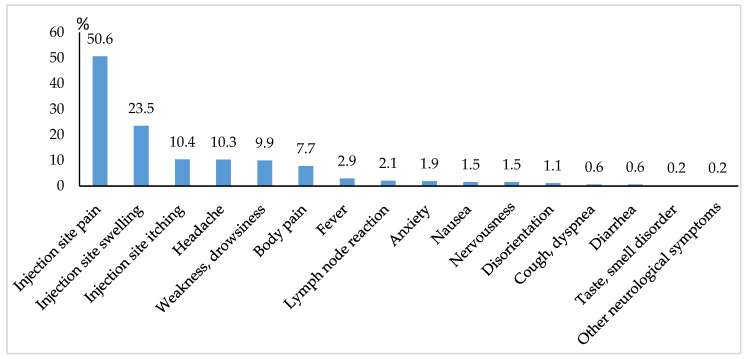
Frequency of adverse events by symptoms.

Adverse events after vaccination were categorized as mild, moderate or severe. The group of mild events included local and systemic reactions (listed in Figure 1), that were not life-threatening and resolved after a few days. Moderate reactions included disorientation, loss of taste or smell, as well as neurological symptoms. Severe reactions were acute allergic reactions/anaphylaxis, and conditions with the risk of disabling. In addition, participants were asked to indicate their age, sex and the result of a qualitative rapid IgG test if one was taken. A total of 5426 questionnaires were collected. Damaged and incomplete questionnaires were removed; however, if vaccine recipients indicated that they experienced adverse events, but did not rate their intensity, such questionnaires were included in the analysis assigning by default unrated answers as 1 (very mild symptoms). Thus, 4181 complete questionnaires were included in the analysis.

### 2.3. Data Processing

Statistical analysis was undertaken using IBM SPSS Statistics^®^ (Statistical Package for Social Sciences 27 for Windows ((IBM, Chicago, IL, USA)). Categorical data were compared using the chi-square or Fisher exact test. The comparison of proportions between groups was performed using the z test. For quantitative data, the nonparametric Mann-Whitney criterion (for two groups) and the Kruskal-Wallis ranking criterion (for three or more groups) were used for comparison. Analysis was performed by age group (<30, 30–46, 47–56 and ≥57 years according to the quartiles that were 30, 47 and 57 years, respectively), sex, experience of adverse events (binary variable, yes or no), use of medication and/or ability to work after vaccination (binary variable, yes or no), experience of moderate adverse events, referral to hospital due to adverse events (binary variable, yes or no) and IgG status (binary variable, positive or negative). Logistic regression analysis was done, and odds ratios (ORs) with 95% confidence intervals (95% CIs) were computed. A *p* value of <0.05 was considered as statistically significant.

### 2.4. Ethics

The study was approved by the Kaunas Regional Biomedical Research Ethics Committee (permission No. BE-2-43, 5 February 2021). Patient informed consent was waived as this study used an anonymous questionnaire.

## 3. Results

This study analysed the data of 4181 participants who were vaccinated with the first dose of the Pfizer-BioNTech vaccine and arrived to receive the second dose between February and March 2021.

More than half (53.6%) of participants experienced at least one adverse event to the vaccine; however, the majority of adverse events were local and systemic reactions (Table 1).

The majority of adverse events occurred during the first two days after vaccination. The most frequent complaint was pain at the injection site (Figure 1).

Moderate adverse events occurred in 1.4% (*n* = 59) of the participants with the majority (*n* = 40) of them being disorientation. The loss of smell or taste and peripheral neurological symptoms (paresthesia) occurred in 10 and 9 participants, respectively.

Comparison by different age groups showed that younger persons were more likely to experience mild and moderate adverse events after vaccination, i.e., with an increasing age, adverse events occurred less frequently (*p* < 0.05) (Table 2). It can be seen that 77.4% of vaccine recipients younger than 30 years experienced adverse events, while this percentage among participants older than 57 years was 31.9%.

**Figure 2:**
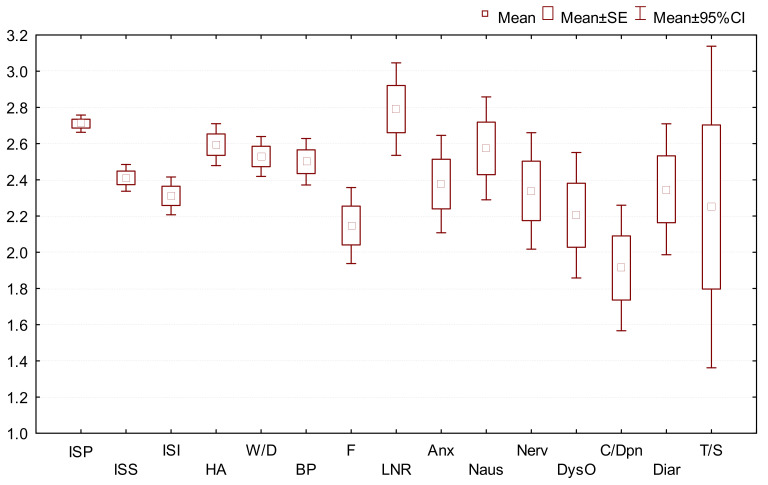
Average score of adverse reaction intensity. ISP–injection site pain; ISS–injection site swelling; ISI–injection site itching; HA–headache; W/D–weakness, drowsiness; BP–body pain; F–fever; LNR–lymph node reaction; Anx–anxiety; Naus–nausea; Nerv–nervousness; DysO–disorientation; C/Dpn–cough, dyspnoea; Diar–diarrhoea; T/S–loss of smell or taste.

The mean scores (rating from 1 to 5) for intensity of all adverse events were similar. The mean score was 2.23 (Figure 2).

Adverse events occurred 1.3 times more frequently in women than men (56.6% vs. 43.5%, *p* < 0.001) (Table 3). The same tendency was observed while evaluating the need for medical care due to adverse events.

Of the 3439 participants who performed a rapid SARS-CoV-2 IgG/IgM test, 3249 (94.5%) were positive for IgG antibodies against SARS-CoV-2 21 days following the first vaccine dose; none showed IgM-specific antibodies. The results of 22 tests were inconclusive, and 168 vaccine recipients were negative for IgG antibodies. Positive anti-SARS-CoV-2 IgG results were more common in participants who experienced adverse events comparing with those who did not experience them (97.1% vs. 92.4%, *p* = 0.02).

As many as 2.6% of the vaccine recipients were referred to a healthcare institution for medical care due to adverse events. Participants who reported more than 3 adverse reactions as well as those with higher intensity scores were more likely to be incapacitated. Women were more likely to refer to a healthcare institution than were men (2.9% vs. 1.6%, *p* = 0.02) (Table 4). Those who referred to a healthcare institution due to adverse events were significantly younger than those who did not seek medical care (*p* < 0.001).

Vaccine efficacy for seroconversion in younger individuals was greater compared with other age groups (Table 5). Being younger than 30 years, aged 30–46 and 47–56 years were respectively associated with a 2.99-fold (95% CI 1.85–4.81, *p* < 0.001), 2.27-fold (95% CI 1.48–3.47, *p* < 0.001) and 1.52-fold (95% CI 1.03–2.26, *p* = 0.036) greater likelihood of having positive results for IgG compared with those aged 57 years and more.

Comparison of the mean intensity scores of all adverse events showed that women scored significantly higher compared with men (Table 6). Moreover, those with positive IgG results from the rapid SARS-CoV-2 test scored significantly higher than participants with negative results for specific IgG (*p* = 0.003).

## 4. Discussion

To our knowledge, this study is the first in the Baltic countries to report data on safety and immunogenicity after the first dose of mRNA-based COVID-19 vaccine in a large cohort of healthcare workers. Our study showed that 53.6% of the participants, especially younger individuals, experienced at least one adverse event after the first vaccine dose. The most frequent adverse events were local reactions at the injection site such as pain (50.6%) and swelling (23.5%).

The findings documented in our study on the adverse events experienced and their incidence are in line with official information from the Centers for Disease Control and Prevention (CDC) [10] and the results of other studies [11,12]. The CDC reported the most common local reactions including pain, redness, swelling at the injection site and general adverse events such as tiredness, headache, muscle pain, chills, fever and nausea. In the study by Zhu et al., these solicited adverse reactions were documented in 72% of participants. The most common systemic reactions were fatigue (42%), fever (32%) and headache (29%) [12]. The study by Baden et al. reported solicited adverse events at the injection site in 84.2% of participants after the first vaccine dose [13].

It is possible that some of the side effects reported could have a psychological origin. The study by Zhu et al. showed that 37% of participants in the placebo group reported adverse events after receiving a physiological solution, with the most common adverse event being fatigue (17%) followed by headache (13%) and pain at the injection site (9%) [12].

In our study, moderate adverse events occurred extremely rarely: only 1.4% of participants experienced disorientation, loss of smell or taste, and other neurological symptoms. No serious adverse reactions (acute allergic reactions/anaphylaxis, conditions with the risk of disabling) were documented. A previous study reported similar results with the most common adverse reactions being mild or moderate [12].

Sometimes it is hard to determine a link with a possible adverse event, because additional knowledge is needed to rule out other potential etiologies. Therefore, complete information on patients’ previous medical and family history should be obtained [14]. This could help identify patients that are at a higher risk of adverse events after vaccination.

We did not document any severe allergic reactions after the first vaccine dose in the large cohort of 4181 individuals. In general, data also show that anaphylactic reactions to vaccinations are extremely rare, occurring at a rate of about 1 per million [15]. A recent report by the CDC indicated that anaphylaxis occurred at a rate of 11.1 cases per million doses administered after vaccination with the Pfizer-BioNTech vaccine [16].

Our study contributes to general understanding of the safety of the Pfizer-BioNTech vaccine during the COVID-19 pandemic. Such studies are of great importance because they fill the information gap between evidence-based and official data. According to the State Medicines Control Agency of Lithuania, as of 31 March 2021, reports of possible adverse events made up 0.34% of the overall number of vaccinations (1742/494,738). One month later, this percentage had decreased to 0.27% (2579/955,921) [7]. Thus, a big difference was observed compared with the data obtained from previous trials and studies. Similar data have been obtained in the United States [16]. As of 23 December 2020, a total of 1,893,360 first doses of Pfizer-BioNTech COVID-19 vaccine had been administered in the USA, and only 0.2% of the vaccine recipients (*n* = 4393) submitted reports of adverse events after vaccination to the Vaccine Adverse Event Reporting System [16].

An important finding of our study is that the percentage of vaccine recipients incapacitated after the first vaccine dose was not considerable. Only 6.2% experienced symptoms that impaired their ability to work. The population of our study was comprised of hospital employees; therefore, it is of high importance that vaccination generally should impair the ability to work to a much lesser degree than the SARS-CoV-2 infection itself.

Our study showed significant vaccine efficacy, triggering the production of IgG antibodies in 94.5% of participants who agreed to perform a rapid IgG test. This is a good result for immunity after the first dose of mRNA vaccine. A study in Croatia showed significant correlation between specific IgG levels after the first dose of the vaccine and six months after full vaccination, regardless of the history of COVID-19. So this indicator may have prognostic value in developing vaccination recommendations for some groups of individuals. However, further research is needed to elucidate the relationship between specific IgG and infection protection [17].

In our study, the percentage of vaccine recipients that tested positive for IgG in rapid SARS-CoV-2 testing was lower among participants aged over 57 years compared with those aged 18 to 46 years. Similar results indicating that older individuals were less immunogenic to mRNA vaccines have also been reported in clinical trials [18]. Milder serologic responses in older people were in parallel with milder systemic reactogenicity: only one third of participants older than 57 years experienced adverse events, and two thirds of individuals aged from 18 to 46 years did not report any adverse events.

Higher rates of specific serologic response were observed in participants who experienced adverse reactions. Moreover, the overall mean score for the intensity of adverse events was greater among vaccine recipients in the positive seroconversion group. This observation could serve as encouragement for people before vaccination, explaining to them that mild systemic reactions are a sign of immune response. Although we know nothing about possible long-term consequences of these novel mRNA-based vaccines [19], for community wellbeing, the proven benefits of vaccinations outweigh the potential risks. One of the limitations of our study was that we were unable to take into account possible long-term adverse reactions after vaccination.

There are some other limitations of this study that should also be mentioned. First, not all participants who filled in the questionnaire also performed a serological test. Second, we reported the data on adverse events and seroconversion rates only after the first vaccine dose and did not include any data about vaccine-induced humoral and cellular immunity, or frequency of adverse events after the second dose. Recall bias is also an important limitation of the study.

Strengths of our study include a large sample size of healthcare workers. Moreover, to our knowledge, this is the first study of its kind in the Baltic region and contributes to the understanding of immunogenicity and frequency of adverse events after the first dose of mRNA vaccine. The data from our study could be used to inform communities that do not support or trust vaccination on the likelihoods of adverse events based on age and sex.

## 5. Conclusions

Our study showed that 53.6% of the participants experienced at least one adverse event after the administration of the first mRNA vaccine dose, and that these were mainly local reactions. Despite 1.4% of subjects indicating moderate adverse events, no safety concerns or anaphylaxis were identified. The first dose of Pfizer-BioNTech vaccine after 21 days induced an immune response, based on rapid SARS-CoV-2 test results in the overwhelming majority (94.5%) of recipients after a single dose. Younger participants experienced more adverse events than their older counterparts and were more frequently positive for anti-SARS-CoV-2 IgG after a single dose of the BioNTech vaccine. It is important to mention that this study specifically considered short-term safety and reactions following vaccination, and long-term adverse effects were not investigated in this study. Thus, future research into both long-term adverse reactions and immune system programming is essential.

## Figures and Tables

**Table 1:** Characteristics of study participants and experienced adverse events in the first 21 days after the first dose of Comirnaty (Pfizes-BioNtech) vaccine.

Characteristic (*n* = 4181)	Value ^1^
Age, mean (SD), years	44.82 (14.1)
Gender	
Female Male	3220 (77.0) 961 (23.0)
Experienced adverse events	2241 (53.6)
Sought medical care due to adverse events	109 (2.6)
Took medication and felt unable to work due to an adverse event	415 (9.9)
Took medication due to an adverse event Felt unable to work due to an adverse event	229 (5.5) 260 (6.2)
Experienced moderate adverse events (disorientation, loss of taste or smell, neurological symptoms)	59 (1.4)

^1^ Values are number (percentage) unless indicated otherwise.

**Table 2:** Incidence of adverse events by different age groups.

Age Group by Years	Total *n*	Experienced Adverse Events *n* (%)	Did not experience Adverse Events *n* (%)	*p*
<30 30–46 47–56 ≥57	961 1121 1019 1080	744 (77.4) 672 (59.9) 480 (47.1) 345 (31.9)	217 (22.6) 449 (40.1) 539 (52.9) 735 (68.1)	<0.001
Total	4181	2241 (53.6)	1940 (46.4)	

**Table 3:** Characteristics of participants who experienced and did not experience adverse events, took or did not take medication, and/or felt or did not feel unable to work, and experienced or did not experience moderate adverse events.

Characteristic	Experienced AE *n* = 2241	Did Not Experience AE *n* = 1940	*p*	Took Medication and/or Felt Unable to Work Due to AE *n* = 415	Did Not Take Medication and/or Did Not Feel Unable to Work Due to AE *n* = 3766	*p*	Experienced Moderate AE *n* = 59	Did Not Experience Moderate AE *n* = 4122	*p*
Female (*n* = 3220) Male (*n* = 961)	1823 (56.6) 418 (43.5)	1397 (43.4) 543 (56.5)	<0.001	375 (11.6) 40 (4.2)	2845 (88.4) 921 (95.8)	<0.001	50 (1.6) 9 (0.9)	3170 (98.4) 952 (99.1)	0.155
Age, mean (SD), years	40.4 (13.5)	50.0 (13.1)	<0.001	38.8 (13.4)	45.5 (14.1)	<0.001	39.5 (13.6)	44.9 (14.1)	0.003
Anti-SARS-CoV-2 IgG+ ^1^	1885 (97.1)	1364 (92.4)	<0.001	352 (98.3)	2897 (94.7)	0.003	53 (100)	3196 (95.0)	0.095

Values are number (percentage) unless indicated otherwise; AE–adverse event; ^1^ rapid SARS-CoV-2 IgG/IgM test result.

**Table 4:** Characteristics of participants who referred or did not refer to a healthcare institution due to adverse events.

Characteristic	Referred *n* = 109	Did Not Refer *n* = 4072	*p*
Female (*n* = 3220) Male (*n* = 961)	94 (2.9) 15 (1.6)	3126 (97.1) 946 (98.4)	0.02
Age, mean (SD), years	39.7 (12.8)	45.0 (14.2)	<0.001
Anti-SARS-CoV-2 IgG+ ^1^	91 (96.8)	3158 (95.0)	0.433

Values are number (percentage) unless indicated otherwise; ^1^ rapid SARS-CoV-2 IgG/IgM test result.

**Table 5:** Characteristics of participants by age, sex and immune response.

Characteristic	*N*	IgG− *n* = 168	IgG+ *n* = 3249	*p*
Age group, years				
<30 30–46 47–56 ≥57	850 923 817 827	24 (2.8) 34 (3.7) 44 (5.4) 66 (8.0)	826 (97.2) 889 (96.3) 773 (94.6) 761 (92.0)	<0.001
Sex				
Female Male	2668 749	122 (4.6) 46 (6.1)	2546 (95.4) 703 (93.9)	0.079

Values are number (percentage) unless indicated otherwise.

**Table 6:** Comparison of the mean intensity scores of all adverse reactions by age, sex and immune response.

Characteristic	*N*	Score, Mean (SD)	*p*
Age group, years			
<30 30–46 47–56 ≥57	737 669 476 342	2.23 (0.86) 2.20 (0.88) 2.26 (0.88) 2.21 (0.89)	0.603
Sex			
Female Male	1809 415	2.30 (0.88) 1.9 (0.80)	<0.001
Anti-SARS-CoV-2: ^1^			
IgG− IgG+	56 1870	1.99 2.26	0.003

^1^ rapid SARS-CoV-2 IgG/IgM test result.

## Data Availability

Not applicable.
